# Editorial: Building a learning health system in pediatric rheumatology

**DOI:** 10.3389/fped.2025.1582988

**Published:** 2025-03-12

**Authors:** Esi M. Morgan, Constance A. Mara, Sheetal S. Vora, Beth S. Gottlieb

**Affiliations:** ^1^ Division of Rheumatology, Department of Pediatrics, Seattle Children’s Research Institute, University of Washington, Seattle, WA, United States; ^2^ Department of Pediatrics, Behavioral Medicine and Clinical Psychology, Cincinnati Children’s Hospital Medical Center, University of Cincinnati College of Medicine, Cincinnati, OH, United States; ^3^ Department of Pediatrics, Atrium Health Levine Children’s Hospital and Wake Forest School of Medicine, Charlotte, NC, United States; ^4^ Department of Pediatrics, Cohen Children’s Medical Center and Northwell, New Hyde Park, NY, United States

**Keywords:** learning health system, rheumatology, pediatrics, quality improvement, chronic disease, electronic health records, patient reported outcome measures, patient engagement

**Editorial on the Research Topic**
Building a learning health system in pediatric rheumatology

Pediatric rheumatic diseases are rare, immune-mediated illnesses that often have a chronic course. Affected children may face a lifetime of challenges including potential disability, persistent pain, and the long-term impact of medications and medical interventions. Early diagnosis and prompt treatment by pediatric rheumatologists using shared decision making can reduce future morbidity. However, many children lack access to timely specialty care due in part to a critical pediatric rheumatology provider workforce shortage, and also limited societal awareness of autoimmune diseases in children resulting in delayed recognition and referral. This vulnerable population of children is left underserved and with their health outcomes uncertain.

In this context, the learning health system (LHS) model offers a promising framework for improving healthcare delivery in pediatric rheumatology (1). A LHS integrates clinical data with continuous quality improvement (QI) and implementation science to enhance outcomes, generate knowledge, and foster research. A community focused on outcomes improvement and a culture of active knowledge sharing is foundational to the success of a LHS. With an estimated 300,000 children in the U.S. affected by rheumatic diseases (2), access to care remains inequitable. The affected population is dispersed across urban and rural settings and is served by pediatric specialists employed at select academic medical centers nationwide, where cognitive subspecialties caring for children are afforded few financial resources. In rare diseases, a shared clinical registry is essential to aggregate sparse data, conduct meaningful analyses, and drive improvements. The LHS model's ability to address health care delivery gaps, drive outcomes improvement, and advance clinical care and knowledge makes it particularly relevant in pediatric rheumatology.

To explore the impact of LHS in this field, we put out a call for a special Research Topic on “Building a Learning Health System in Pediatric Rheumatology”. Our goal was to highlight real-world clinical practice and network interventions to improve the quality of healthcare delivery and outcomes in pediatric specialty care. The editorial team is pleased to highlight a broad overview of the 12 articles selected by peer review for publication, representing 23 distinct health systems across North America.

Key themes reflecting drivers of improving chronic illness care emerged across these contributions. A study by Vora et al. addressed delays in referral and low access to care. The authors describe a single center intervention to increase timely referrals to rheumatology care from safety net primary care clinics through education, raising awareness of presenting symptom bundles of rheumatic diseases, streamlining referral processes, and establishing a triage system with expedited scheduling for urgent cases. This work exemplifies the capability of a single clinic to influence access and timeliness of care within the confines of a larger health system.

Another major theme was addressing variation in treatment practices. Balay-Dustrude et al. describes unwarranted variation in care in a then nascent learning health network (Pediatric Rheumatology Care and Outcomes Improvement Netowrk, PR-COIN), through evaluation of use of intra-articular corticosteroid injections (IACI) following release of American College of Rheumatology JIA treatment guidelines (in 2011, 2013). Data from 2011 to 2015 was analyzed on whether IACI were used as primary treatment for oligoarticular JIA. Although there was no network-level intervention, centers worked locally to try and achieve a network stated goal of IACI within 2 weeks of diagnosis. Despite local efforts, results revealed lower-than-expected IACI rates and regional practice variation, highlighting the need for standardized care approaches within a LHS.

Multiple articles touched on the importance of data capture to assess patient outcomes to evaluate for potential gaps in care or disparities, and of the reliable collection of disease specific activity measures across patient population. Goh et al., and Pan et al., conducted surveys of PR-COIN LHS members to understand current state of data collection across network centers, and understand barriers and facilitators to reliable collection. Goh focuses on the role of ‘critical data elements’ and offers unique insights on the challenge of collection during tele-rheumatology visits. Patient centered care requires assessment of outcomes directly from the patients (PROs), Pan shares best practices for systematic PRO collection based on network experience. Although health equity is a key principle of quality of care, a fundamental prerequisite is data completeness which confers the ability to identify disparities. Banschbach et al., through an evaluation of PR-COIN learning network registry data identified missing demographic data in 1/3 of patients. With interventions, this was remediated in about 94%. The article noted that patients missing race data are likely to be missing other critical data elements, and challenges us to equitably measure disease activity and ensure data capture for vulnerable groups.

Two studies detailed single-center interventions leveraging electronic health records (EHR) for data-driven improvement. Timmerman et al. and Barbar-Smiley et al., each provide single center interventions to standardize disease activity data collection in the electronic health record (EHR). Timmerman describes partnership with local IT resources to establish a center dashboard to capture data on all clinic patients to support QI initiatives. Barbar-Smiley's group leveraged features within the EHR collection system to support and sustain a clinic effort to capture systemic lupus (SLE) disease activity, a process that also benefitted their research registry participation.

Huang et al. describe use of technology to leverage clinical registry data of an LHS to refine algorithms supporting selection of personalized treatment based on real world experience of other similar patients, which can be used at point of care in shared decision making with patients.

Mental health and self-management support were also focal points. Harper et al. present an intervention to systematically collect mental health data in childhood SLE patients, and an intervention to respond with mental health supports. Argraves et al., address the perpetual challenge of assessing and supporting transition readiness in children reaching age of majority. They leverage process automation to achieve reliability in assessment (90%). The intervention also requires provider review and self-management support education. That multiple QI interventions were published from one center suggests a strong culture of QI, and effective use of QI as a strategy for operational improvement.

Two articles highlighted outcomes improvement and parent engagement within PR-COIN, a pediatric rheumatology LHS with over 7,200 active patients across 23 centers. Harris et al. and Ferraro et al., discuss network-wide interventions and outcome improvements demonstrating the effectiveness of the LHS model in driving sustainable improvements. Of vital importance to the network's success is the engagement of parents and patients in design and conduct of interventions contributing the patient perspective, and keeping focus on patient outcomes.

What can we take away from this issue? The inspiration of pediatric rheumatology clinical teams working in coordination together with parents to move the needle on healthcare improvement for a vulnerable patient population. A culture of QI, rigorous data collection, and the use of technology are central to this work. By focusing on equitable access, timely diagnosis and treatment, mental health integration, proactive care teams, engaged families, and health system support for QI, a LHS organizational framework has been shown to improve processes and patient outcomes in pediatric rheumatology. This special issue serves as a testament to the power of adopting a LHS model in transforming care in pediatric chronic illness care.
